# Using glucagon receptor antagonism to evaluate the physiological effects of extrapancreatic glucagon in totally pancreatectomised individuals: a randomised controlled trial

**DOI:** 10.1007/s00125-025-06534-z

**Published:** 2025-09-18

**Authors:** Caroline Trunk-Black Juel, Asger B. Lund, Sofie Hædersdal, Maria M. Andersen, Carsten P. Hansen, Jan H. Storkholm, Gerrit van Hall, Bolette Hartmann, Mette M. Rosenkilde, Camilla J. Kibsgaard, Flemming Dela, Nicolai J. Wewer Albrechtsen, Jens J. Holst, Tina Vilsbøll, Filip K. Knop

**Affiliations:** 1 Center for Clinical Metabolic Research, Copenhagen University Hospital – Herlev and Gentofte, Hellerup, Denmark; 2 Department of Clinical Medicine, Faculty of Health and Medical Sciences, University of Copenhagen, Copenhagen, Denmark; 3 Copenhagen University Hospital – Steno Diabetes Center Copenhagen, Herlev, Denmark; 4 Department of Surgery, Copenhagen University Hospital - Rigshospitalet, Copenhagen, Denmark; 5 Department of Clinical Biochemistry, Copenhagen University Hospital - Rigshospitalet, Copenhagen, Denmark; 6 Clinical Metabolomics Core Facility, Copenhagen University Hospital - Rigshospitalet, Copenhagen, Denmark; 7 Department of Biomedical Sciences, Faculty of Health and Medical Sciences, University of Copenhagen, Copenhagen, Denmark; 8 Department of Geriatrics, Copenhagen University Hospital - Bispebjerg and Frederiksberg, Copenhagen, Denmark; 9 Novo Nordisk Foundation Center for Basic Metabolic Research, Faculty of Health and Medical Sciences, University of Copenhagen, Copenhagen, Denmark; 10 Novo Nordisk A/S, Søborg, Denmark

**Keywords:** Glucagon, Glucagon receptor antagonist, LY2409021, Postprandial glucose excursions, Total pancreatectomy

## Abstract

**Aims/hypothesis:**

Previous studies have indicated that 29-amino-acid glucagon (i.e. ‘pancreatic’ glucagon) circulates in totally pancreatectomised individuals and that a postprandial glucagon response can be detected. Using a glucagon receptor antagonist (GRA), we investigated the possible role of extrapancreatic glucagon on glucose, lipid and amino acid metabolism in totally pancreatectomised individuals.

**Method:**

In a randomised, crossover study, nine totally pancreatectomised individuals and nine matched healthy control individuals were given, in randomised order (planned on the website www.random.org), 300 mg GRA (LY2409021; Eli Lilly) or placebo 10 h before two 3 h OGTTs. The experiment was double-masked (i.e. both participants and investigator were masked for the type of the experimental day [day A vs day B]). The key inclusion criteria for the healthy control participants were age >18 years, normal fasting plasma glucose and HbA_1c_ 31–44 mmol/mol (5.0–6.2%), haemoglobin >7.0 mmol/l (men) / >6.5 mmol/l (women) and informed consent. Key inclusion criteria for the pancreatectomised individuals were age >18 years, haemoglobin in the normal range and informed consent. The primary endpoint was the difference in plasma glucose excursions between study days.

**Results:**

Glucagon concentrations remained unchanged from fasting concentrations during the OGTT in the totally pancreatectomised individuals on both study days and circulating glucose, lipids and amino acid levels were unaffected by treatment with LY2409021 compared with placebo. In the control group, LY2409021 resulted in relevant pharmacodynamic effects, including lower fasting plasma glucose (4.7 [0.1] vs 5.2 [0.1] mmol/l, *p*=0.001) and augmented concentrations of amino acids in plasma, compared with placebo.

**Conclusions/interpretation:**

We conclude that inhibition of the glucagon receptor using LY2409021 during OGTT in totally pancreatectomised individuals does not produce detectable effects on glucose, lipid or amino acid metabolism, ruling out metabolic effects of extrapancreatic glucagon.

**Trial registration:**

ClinicalTrials.gov (NCT02944110).

**Funding:**

This study was supported by grants from the Aase and Ejnar Danielsen’s Foundation and the Novo Nordisk Foundation.

**Graphical Abstract:**

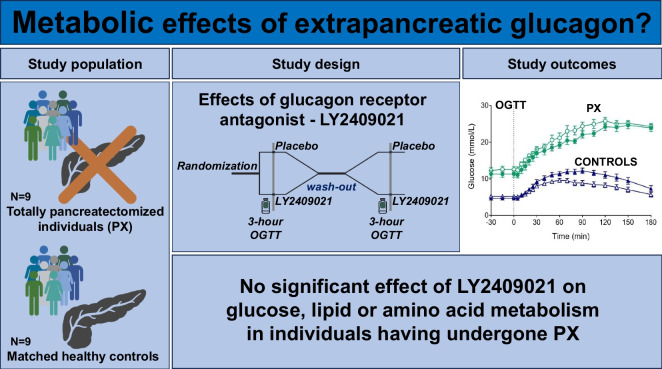

**Supplementary Information:**

The online version of this article (10.1007/s00125-025-06534-z) contains peer-reviewed but unedited supplementary material.


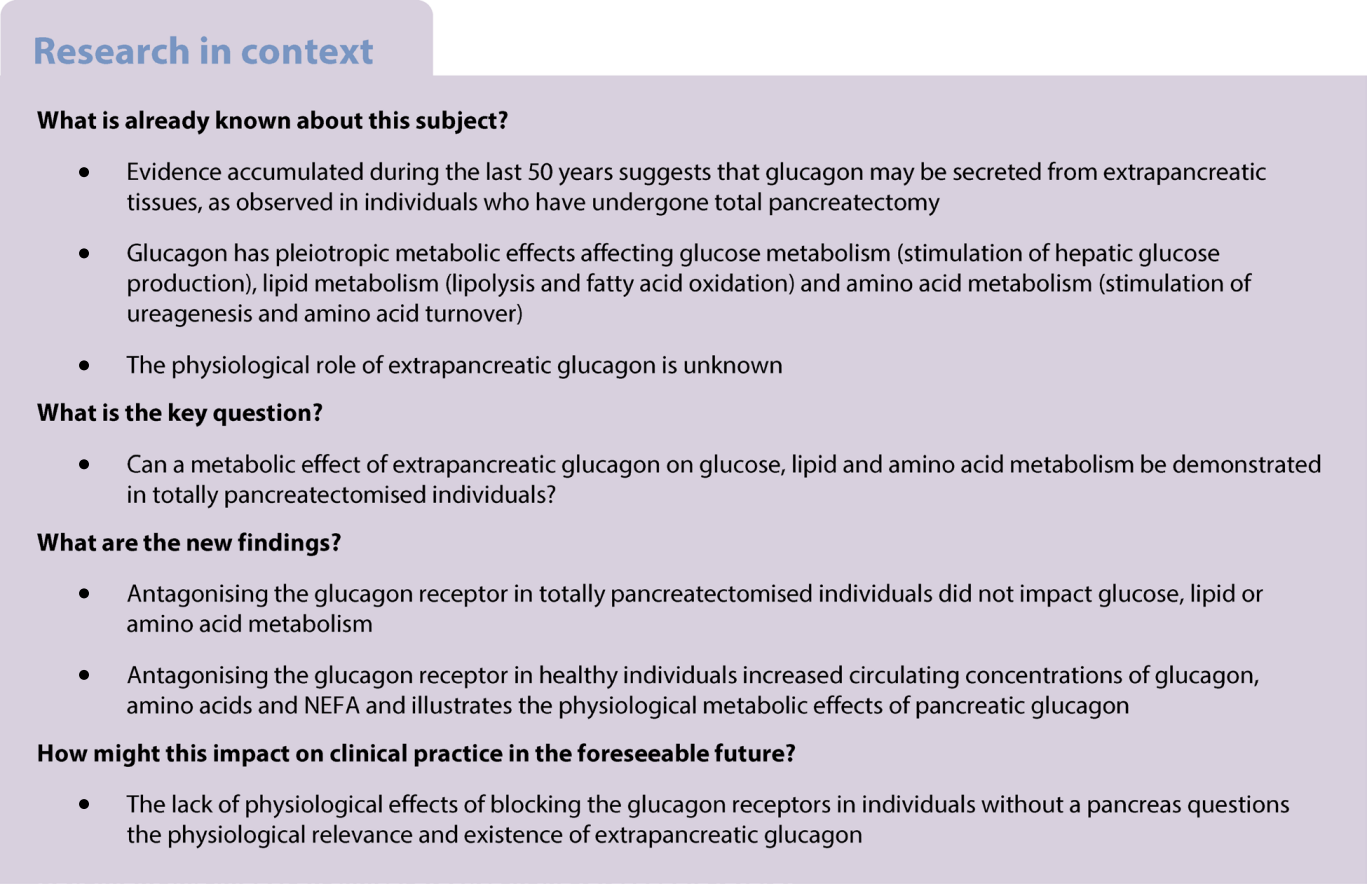


## Introduction

Glucagon is a 29-amino-acid peptide hormone derived from the precursor proglucagon and is generally perceived as a pancreas-specific hormone secreted from alpha cells in the islets of Langerhans [1]. The main target of glucagon is the liver and the most thoroughly described hepatic effect of glucagon is its stimulation of hepatic glucose production (via stimulation of gluconeogenesis and glycogenolysis) [2, 3]. Glucagon is also known to be a powerful regulator of amino acid metabolism, increasing hepatic amino acid turnover by stimulating ureagenesis and gluconeogenesis, which in turn decrease plasma amino acid concentrations [4]. Circulating amino acids stimulate glucagon secretion from pancreatic alpha cells [5, 6] and, thus, circulating concentrations of glucagon and amino acids regulate each other via a feedback loop, the liver–alpha cell axis [7–9]. Moreover, glucagon is known to reduce the fat content in the liver via stimulation of lipolysis and fatty acid oxidation and via inhibition of lipogenesis [10]. Finally, glucagon administered by the s.c, i.m. or i.v. route in supraphysiological doses has been shown to reduce appetite and food intake [11] and increase resting energy expenditure (REE) [12]; however, the role of endogenous glucagon in these processes remains unknown.

Evidence accumulated during the last 50 years suggests that glucagon may also be secreted from the gastrointestinal tract, as observed in individuals who have undergone total pancreatectomy, indicating that cells other than pancreatic alpha cells may be able to secrete glucagon [13–15]. Small intestinal biopsies from these individuals seem to contain extractable glucagon in concentrations that are higher than those found in similar biopsies from matched healthy control individuals [16]. The physiological effects of any extrapancreatic glucagon are currently unknown.

In the present study, we administered a glucagon receptor antagonist (GRA) to totally pancreatectomised individuals and to matched healthy individuals to evaluate the effects of extrapancreatic glucagon secretion during OGTT on glucose, amino acid and lipid metabolism.

## Methods

### Endpoints

The primary endpoint was the difference in plasma glucose excursions between test days with and without a GRA (LY2409021) in totally pancreatectomised individuals as assessed by baseline-subtracted AUC (bsAUC) from time 0 to 180 min during a 75 g OGTT. Secondary endpoints were differences in glucose and glycerol kinetics, circulating levels of C-peptide, glucagon, NEFA, triglycerides, cholesterols, amino acids, glucose-dependent insulinotropic polypeptide (GIP) and glucagon-like peptide 1 (GLP-1), BP, appetite sensations, food intake, gastric emptying, diuresis and REE after intake of LY2409021 and placebo.

### Conduct, registration and approvals

The study was conducted at the Center for Clinical Metabolic Research, Gentofte Hospital, University of Copenhagen, Hellerup, Denmark, in accordance with the Helsinki Declaration, registered at ClinicalTrials.gov (NCT02944110) and approved by the Ethics Committee of the Capital Region of Denmark (H-15009763).

### Study participants

**Fig. 1:**
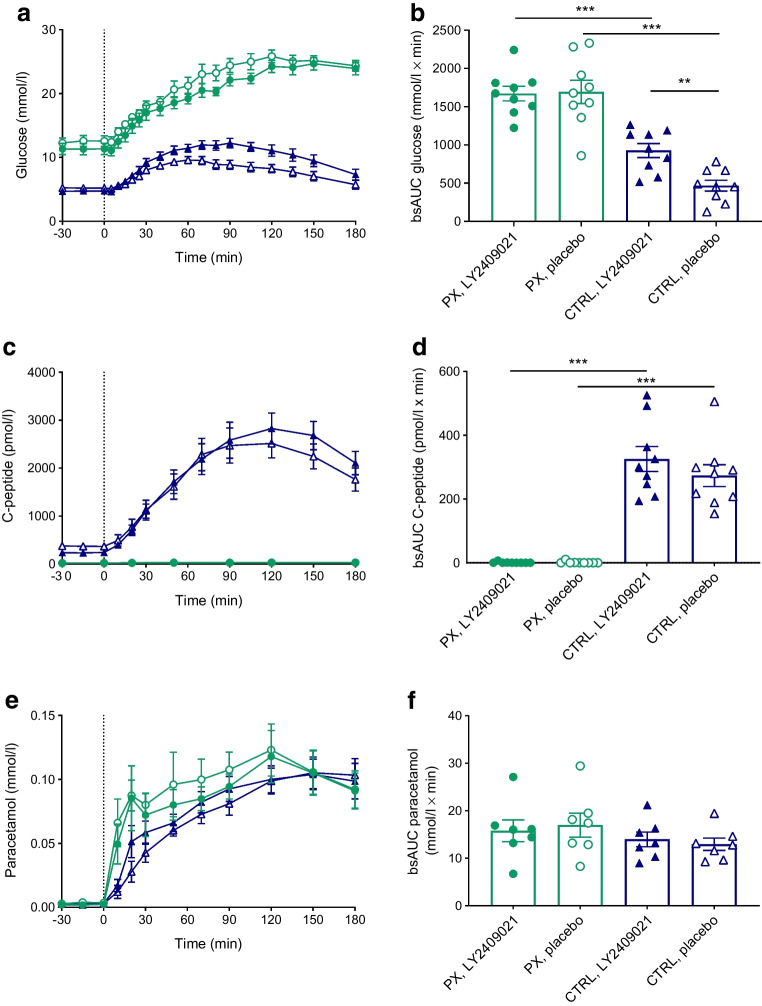
Plasma glucose (**a**) C-peptide (**c**) and paracetamol (**e**) concentrations during a 75 g OGTT (initiated at time 0 min) in nine totally pancreatectomised participants (PX) (green circles) and nine control participants (CTRL) (blue triangles) with the GRA LY2409021 (filled symbols) or placebo (open symbols). bsAUC values are also shown (**b**, **d**, **f**). Data are shown as means ± SEM. **p<0.01, ***p<0.001

We included nine totally pancreatectomised participants (seven men and two women) and nine matched healthy control participants. Matching criteria were age ±20% and BMI ±20%. Due to limited opportunities for inclusion by the small number of available pancreatectomised individuals, sex distribution is not integrated in the study design. The key inclusion criteria for the healthy control participants were age >18 years, normal fasting plasma glucose and HbA_1c_ 31–44 mmol/mol (5.0–6.2%), haemoglobin >7.0 mmol/l (men) / >6.5 mmol/l (women) and informed consent. Sex was self-reported by participants, options were woman or man. Key inclusion criteria for the pancreatectomised individuals were age >18 years, haemoglobin in the normal range and informed consent. Key exclusion criteria for both groups were age >80 years, inflammatory bowel disease and severe liver and/or kidney disease (electronic supplementary material [ESM] Fig. 1). For the total pancreatectomy group, additional exclusion criteria were pancreatectomy within the last 3 months and ongoing chemotherapy or chemotherapy within the last 3 months.

### GRA

LY2409021 is an orally administered, potent, competitive, small-molecule GRA that binds to and blocks the human glucagon receptor. The median time for maximum drug concentration (t_max_) is 4–8 h after intake and the mean t½ is ~55 h [17, 18]. LY2409021 and LY2409021 placebo were provided cost free by Eli Lilly (Indianapolis, IN, USA).

### Experimental procedures

After an initial screening visit, participants underwent two study days, performed in randomised order within 1 month and with 14 days between the study days for drug washout. Randomisation was performed using www.random.org. Participants were instructed to refrain from strenuous physical exercise and alcohol intake for 2 days prior to the screening visit and the two experimental days. At bedtime before each experimental day (i.e. at ~22:00 hours), participants received 300 mg LY2409021 (day A) or placebo (day B). The experiments were masked for both investigators and participants. A laboratory technician was responsible for single-masked dispense of experimental medicine but otherwise did not take part in the study. The pancreatectomised participants were instructed to take their long-acting insulin the evening before each experimental day and to defer from further insulin dosing until completion of the experimental day. Each experimental day lasted ~7 h. On both experimental days, the participants arrived after an overnight 10 h fast and were placed in a semi-recumbent position in a hospital bed. Cannulas were inserted into a cubital vein of each arm, one for infusion of stable isotopes and one in the other arm for collection of arterialised blood; the hand and forearm were wrapped in a heating pad (~42°C) throughout the entire experimental day. After basal blood specimens had been collected (time −120 min), stable isotope-labelled glucose ([6,6-D_2_]glucose) and glycerol ([1,1,2,3,3-D_5_]glycerol) (Cambridge Isotope Laboratories, Tewksbury, MA, USA) dissolved in saline (154 mmol/l NaCl) were infused intravenously ([6,6-D_2_]glucose at a priming dose of 17.5 µmol/kg × fasting plasma glucose / 5 and a maintenance rate of 0.6 µmol kg^−1^ min^−1^ and [1,1,2,3,3-D_5_]glycerol at a priming dose of 2 µmol/kg and maintenance rate of 0.1 µmol kg^−1^ min^−1^). At time 0 min, participants ingested a 71.5 g glucose solution with 3.5 g [U-^13^C_6_]glucose as a tracer of ingested glucose. Paracetamol (acetaminophen) (1.5 g) was added for evaluation of gastric emptying [19]. Blood samples were drawn at regular intervals from time −30 to 180 min. Further details on experimental procedures are available in ESM Methods.

### Biochemical analysis

Glucose concentrations were measured using the glucose oxidase method (Yellow Springs Instrument Model 2900D Biochemistry Analyzer, Yellow Springs, Ohio, USA). Glucagon concentrations in plasma were measured using a sandwich ELISA (Mercodia, Uppsala, Sweden) based on N- and C-terminal-wrapping monoclonal antibodies, respectively, with a protocol including additional washing steps (the so-called sequential protocol), as described previously [20]. Plasma enrichment of [6,6-D_2_]glucose, [U-^13^C_6_]glucose and [1,1,2,3,3-D_5_]glycerol was determined using LC tandem-MS (LC-MS/MS) [21]. Further details on biochemical analyses are available in the ESM Methods.

### Calculations and statistical analysis

Data are presented as mean (SEM) unless otherwise stated. AUC was calculated using the trapezoid rule and are presented as bsAUC when nothing else is stated. Glucose and glycerol rate of appearance (*R*_a_ glucose, *R*_a_ glycerol) and rate of disappearance (*R*_d_ glucose, *R*_d_ glycerol) were calculated from changes in glucose and glycerol enrichment and a pool fraction of 70 ml/kg using the one-compartment, fixed-volume, non-steady-state model of Steele [22, 23]. To calculate endogenous glucose production (EGP), the *R*_a_ of the ingested glucose was subtracted from the total rates of glucose appearance. Group differences in baseline characteristics and bsAUCs were evaluated using two-sample Student’s *t* test, paired tests within groups and unpaired tests between groups; the latter comparisons were only performed between days with similar interventions (GRA/placebo). Statistical analysis was structured to reflect the crossover design by using paired comparisons between treatment conditions within individuals, and a 14 day washout period was included to minimise potential carry-over effects. *p* values ≤0.05 were accepted as statistically significant. Statistical evaluation and graphic presentation were made in GraphPad Prism 7 (La Jolla, CA, USA).

## Results

### Study participants

Nine totally pancreatectomised individuals (mean [SD]: age 61.3 [3.4] years; BMI 22.6 [1.6] kg/m^2^; HbA_1c_ 68 [8] mmol/mol or 8.4 [0.7]%) and nine matched healthy individuals (mean [SD]: age 65.9 [2.9] years; BMI 23.9 [0.9] kg/m^2^; HbA_1c_ 31 [4] mmol/mol or 5 [0.2]%) with no family history of diabetes were included (Tables 1, 2). All participants in the pancreatectomy group had undergone a total pancreatectomy, duodenectomy and splenectomy performed as a non-pylorus-preserving operation with division of the gastric antrum ~2 cm above the pylorus. All participants gave informed consent for participation. The study flowchart is displayed in ESM Fig. 1.

**Table 1:** Clinical characteristics of the totally pancreatectomised participants

Participant no.	Sex	Time since operation (years)	BMI (kg/m^2^)	Insulin treatment (U)^a^	Reason for surgery	Other treatment
1	Male	6.7	25.2	Insulin glargine 8 + 0 + 10	Adenocarcinoma	Creon 40,000 three times a day plus Creon 10,000 at between-meal snacks
				Insulin aspart 10 + 0 + 10		Pantoprazole 40 mg twice a day
2	Male	2.7	21.4	Insulin detemir 12 + 0 + 0	Adenocarcinoma	Creon 80,000 three times a day plus Creon 25,000 twice a day at between-meal snacks
				Insulin aspart 5 + 5 + 5		Pantoprazole 40 mg twice a day
						Mirtazapine 15 mg once a day
						Citalopram 40 mg once a day
						Magnesium 360 mg once a day
						Loperamide 2 mg once a day
3	Male	6.4	30.3	Insulin detemir 24 + 0 + 14	Neuroendocrine tumour	Creon 25,000 five times a day plus Creon 10,000 at between-meal snacks
				Insulin aspart 7 + 7 + 7		Paracetamol 665 mg six times a day
						Quinine 100 mg twice a day
						Lansoprazole 30 mg once a day
						Vitamin D 25 µg once a day
						Glucosamine 400 mg three times a day
						Oxycodone hydrochloride 5 mg twice a day
4	Female	2.7	17.3	Insulin detemir 9 + 0 + 6	IPMN	Creon 25,000 three times a day
				Insulin aspart 4 + 2 + 2		
5	Female	8.0	16.2	Insulin glargine 12 + 0 + 0	Adenocarcinoma	Creon 25,000 three times a day plus Creon 10,000 at between-meal snacks
				Insulin aspart 7 + 7 + 7		Levothyroxine 30 mg twice a day
						Lansoprazole 30 mg once a day
6	Male	1.4	24.0	Insulin detemir 20 + 0 + 9	Adenocarcinoma	Creon 50,000 three times a day plus Creon 50,000 at between-meal snacks
				Insulin aspart 6 + 6 + 6		Promethazine 25 mg once a day
						Pantoprazole 40 mg twice a day
						Losartan potassium 100 mg once a day
						Bendroflumethiazide 2.5 mg twice a day
						Amlodipine 5 mg once a day
7	Male	4.3	19.5	Insulin detemir 17 + 0 + 7	Pancreatitis	Creon 25,000 three times a day
						Pantoprazole 40 mg twice a day
				Insulin aspart 7 + 4 + 4		Buprenorphine 0.2 mg three times a day
8	Male	0.4	21.6	Insulin detemir 14 + 0 + 6	Pancreatitis	Creon 75,000 three times a day
						Tramadol 50 mg twice a day
				Insulin aspart 2 + 2 + 2		Pantoprazole 40 mg twice a day
						Melatonin 3 mg once a day
9	Male	0.4	28.5	Insulin detemir 15 + 0 + 15	IPMN	Creon 25,000 three times a day plus Creon 25,000 at between-meal snacks
				Insulin aspart 6 + 6 + 6		Bendroflumethazide 2.5 mg once a day
						Amlodipine 10 mg once a day
						Ramipril 10 mg once a day
						Spironolactone 25 mg once a day

Creon contained the following pancreatic enzymes: Creon 10,000 contained 8000 EP-e (enteric-coated pancreatic enzyme units) amylase, 10,000 EP-e lipase and 600 EP-e protease; Creon 25,000 contained 18,000 EP-e amylase, 25,000 EP-e lipase and 1000 EP-e protease; and Creon 40,000 contained 25,000 EP-e amylase, 40,000 EP-e lipase and 1600 EP-e protease

^a^Insulin doses are shown as U taken morning, noon and evening

IPMN, intraductal papillary mucinous neoplasm

**Table 2:** Clinical characteristics of the control participants

Control participant no.	Sex	BMI (kg/m^2^)	Treatment
1	Male	28.2	Fluticasone 50 µg / 500 µg once a day
			Tiotropium bromide 18 mg once a day
2	Male	18.7	-
3	Female	22.5	Zoledronate injection 5 mg once a year
4	Male	23.6	-
5	Male	24.5	-
6	Male	24.2	-
7	Female	22.8	-
8	Male	24.4	Simvastatin 40 mg once a day
			Enalapril 20 mg once a day
9	Female	26.6	-

### Glucose

In the pancreatectomy group, treatment with 300 mg LY2409021 did not change fasting plasma glucose or plasma glucose excursions in response to OGTT compared with placebo (Table 3, Fig. 1a). In the control group, mean fasting plasma glucose concentrations were significantly lower after LY240921 (4.7 [0.1] vs 5.2 [0.1] mmol/l, *p*=0.001) and plasma glucose excursions in response to OGTT were higher (926 [92] vs 467 [72] mmol/l × min, *p*=0.002) (Table 3, Fig. 1a).

**Table 3:** Glucose, hormone and paracetamol levels during OGTT

Variable	Totally pancreatectomised participants			Control participants		
	LY2409021	Placebo	*p* value	LY2409021	Placebo	*p* value
Glucose						
Baseline (mmol/l)	11.3 (0.9)	12.5 (0.8)	0.266	4.7 (0.1)	5.2 (0.1)	0.001
*C*_max_ (mmol/l)	25.4 (1.0)	26.5 (0.9)	0.383	12.6 (0.7)	10.3 (0.5)	0.013
AUC (mmol/l × min)	3708 (156)	3984 (162)	0.179	1776 (97)	1405 (79)	0.008
bsAUC (mmol/l × min)	1671 (96)	1693 (152)	0.810	926 (92)	467 (72)	0.002
C-peptide						
Baseline (pmol/l)	21.7 (5.7)	20.7 (3.4)	0.509	236 (25.5)	370 (58.2)	0.007
*C*_max_ (pmol/l)	28 (10.4)	30.2 (11.7)	0.318	3088 (300)	2842 (293)	0.242
AUC (nmol/l × min)	4.8 (1.8)	4.9 (1.9)	0.199	368 (42)	340 (39.3)	0.286
bsAUC (nmol/l × min)	0.8 (0.7)	1.3 (1.2)	0.332	325 (39.1)	273 (34.5)	0.087
Paracetamol						
*C*_max_ (mmol/l)	0.14 (0.0)	0.14 (0.0)	0.437	0.11 (0.0)	0.10 (0.0)	0.739
AUC (mmol/l × min)	1.7 (0.5)	1.9 (0.3)	0.609	1.1 (0.2)	0.7 (0.2)	0.002
bsAUC (mmol/l × min)	15.8 (2.3)	16.9 (2.5)	0.094	14.1 (1.4)	13.1 (1.1)	0.045
Glucagon						
Baseline (pmol/l)	0.8 (0.0)	0.8 (0.0)	0.256	7.4 (3.1)	1.9 (0.4)	0.079
*C*_max_ (pmol/l)	0.9 (0.1)	1.4 (0.3)	0.082	10.6 (4.2)	2.7 (0.6)	0.072
AUC (pmol/l × min)	138 (1.7)	155 (9.7)	0.095	366 (84)	211 (33)	0.023
bsAUC (pmol/l × min)	−3.3 (3.0)	18.4 (8.9)	0.057	−988 (482)	−129 (51)	0.099
GLP-1						
Baseline (pmol/l)	10.2 (2.0)	10.4 (1.9)	0.946	4.9 (0.7)	4.2 (0.9)	0.487
*C*_max_ (pmol/l)	73.1 (15)	75.8 (10)	0.823	19.0 (2.5)	18.0 (1.9)	0.709
AUC (pmol/l × min)	6030 (1115)	5610 (865)	0.497	2288 (300)	2053 (252)	0.285
bsAUC (pmol/l × min)	4191 (1157)	3730 (855)	0.646	1395 (319)	1306 (316)	0.810
GIP						
Baseline (pmol/l)	18.2 (1.8)	17.2 (1.5)	0.610	12.7 (1.3)	10.6 (1.4)	0.303
*C*_max_ (pmol/l)	103 (10)	96.2 (11)	0.415	78.0 (8.4)	60.3 (6.1)	0.006
AUC (pmol/l × min)	11,988 (1150)	9586 (919)	0.025	10,495(1094)	8274 (921)	0.005
bsAUC (pmol/l × min)	8718 (956)	6485 (729)	0.024	8209 (973)	6361 (893)	0.016

Data are shown as mean (SEM)

Plasma/serum concentrations of glucose, C-peptide, paracetamol, glucagon, GLP-1 and GIP were measured during a 3 h 75 g OGTT with 1.5 g paracetamol in nine totally pancreatectomised participants and nine control participants receiving LY2409021 or placebo

Statistical analysis was performed by two-sample Student’s *t* test (two-tailed), paired within groups and unpaired between groups

*C*_max_, maximum serum/plasma concentration

### Glucose kinetics

**Fig. 2:**
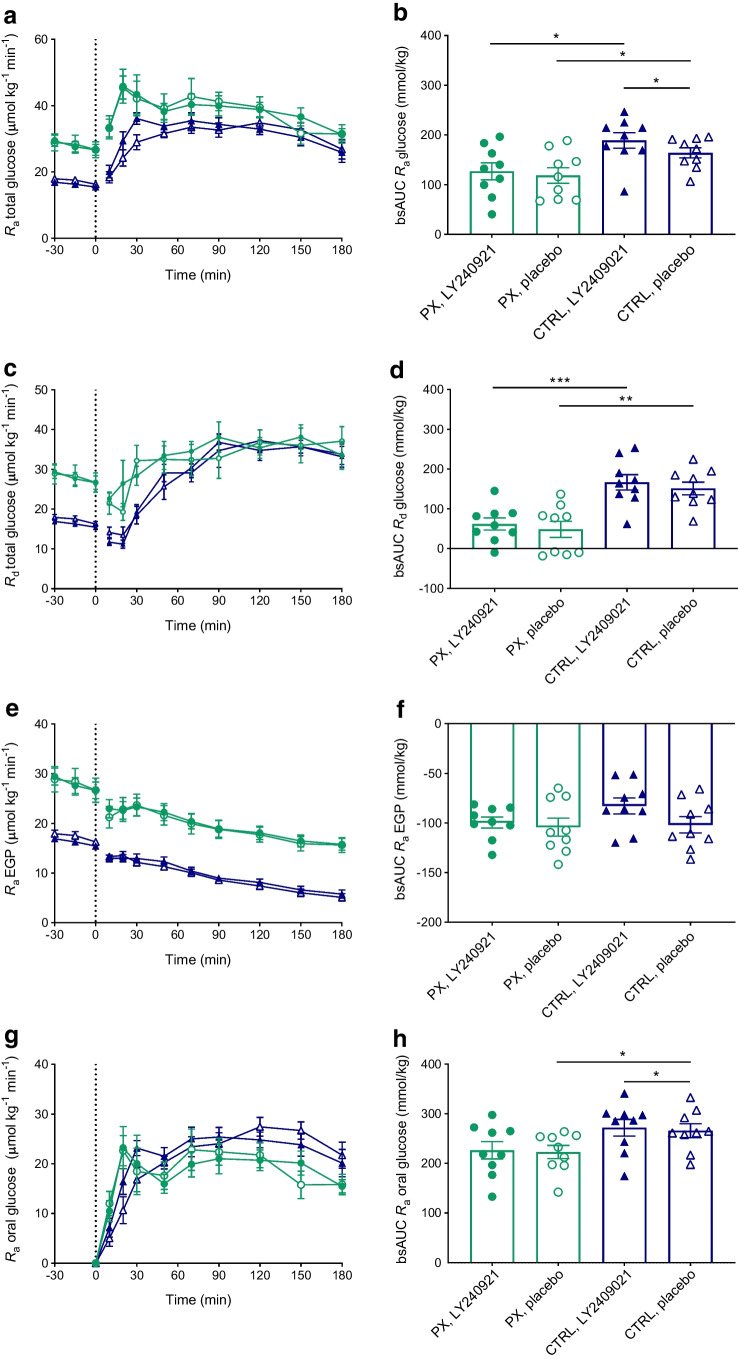
Total glucose *R*_a_ (**a**), total *R*_d_ (**c**), EGP *R*_a_ (**e**) and oral glucose *R*_a_ (**g**) during a 75 g OGTT (initiated at time 0 min) in nine totally pancreatectomised participants (PX) (green circles) and nine control participants (CTRL) (blue triangles) with the GRA LY2409021 (filled symbols) or placebo (open symbols). bsAUCs (**b**, **d**, **f**, **h**) are also shown. Data are shown as means ± SEM. *p≤0.05, **p<0.01, ***p<0.001

In the fasting state, EGP, total *R*_a_ glucose and *R*_d_ glucose were higher in the pancreatectomy group than in the control group (Fig. 2a, c, e). LY2409021 did not cause significant changes in EGP, total *R*_a_ or *R*_d_ glucose during fasting nor during OGTT in either of the two groups (Fig. 2a–f). *R*_a_ oral glucose was similar on the two study days in the pancreatectomy group (Fig. 2g, h). Glucose concentrations in urine reflected plasma levels and were significantly higher in the pancreatectomy group compared with the control group (ESM Table 1). Urinary glucose excretion did not differ between study days in any of the groups.

### C-peptide

In all participants (except one) in the pancreatectomy group, C-peptide concentrations were below the detection limit of the assay (<16 pmol/l) at all time points on both study days. One participant (participant no. 6; operated on due to adenocarcinoma) showed low concentrations of C-peptide activity with a peak value of 129 pmol/l at 120 min (plasma glucose 25.9 mmol/l) on the placebo day. Other endpoints, including glucagon, were not different in this participant compared with the mean group values, so data from this participant were kept in the dataset for further analyses. In the control group, and concomitant with the LY2409021-induced reduction in fasting plasma glucose, fasting concentrations of C-peptide decreased after LY2409021 whereas C-peptide responses to OGTT with LY2409021 and placebo, respectively, were similar (325 [39] vs 273 [35] nmol/l × min,* p*=0.087) (Table 3, Fig. 1c, d).

### Glucagon

**Fig. 3:**
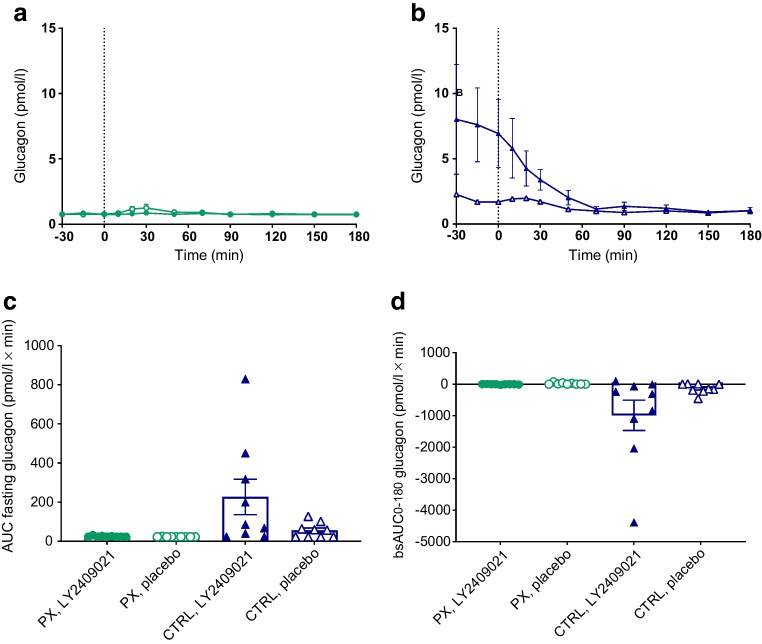
(**a**, **b**) Plasma glucagon concentrations during a 75 g OGTT (initiated at time 0 min) in nine totally pancreatectomised participants (PX) (**a**, green circles) and nine control participants (CTRL) (**b**, blue triangles) with the glucagon receptor antagonist LY2409021 (filled symbols) or placebo (open symbols). (**c**, **d**) AUC values during fasting conditions (**c**) and bsAUC values during OGTT (**d**) are shown. Data are shown as means ± SEM

On placebo days, fasting concentrations of glucagon in the pancreatectomy group were significantly lower than in the control group, and they were unaffected by LY2409021 (Fig. 2 and ESM Table 1). Glucagon concentrations remained unchanged from fasting concentrations during the OGTT in the pancreatectomy group on both study days. In the control group, mean fasting concentrations of glucagon were fourfold higher on the LY2409021 day than on the placebo day but the difference did not reach statistical significance (7.4 [3.1] vs 1.9 [0.4] pmol/l, *p*=0.079). During the OGTT in the control group, a gradual normalisation of the LY2409021-induced hyperglucagonaemia was observed and plasma glucagon concentrations reached normal baseline levels at 90 min (Fig. 3).

### Paracetamol

In the pancreatectomy group, bsAUC and maximum serum/plasma concentration of paracetamol during the OGTTs were similar on the two study days (*p*=0.094 and *p*=0.437) (Table 3, Fig. 1e, f). Data from two participants were excluded because no paracetamol was detected in any of the samples (probably due to lack of paracetamol addition to the OGTT by mistake). In the control group, there was no difference in the overall paracetamol levels between study days. However, immediately after the ingestion of glucose in the OGTT, significantly higher paracetamol concentrations were observed with LY2409021 (AUC_0–20min_, *p*=0.011).

### Lipid profile

**Fig. 4:**
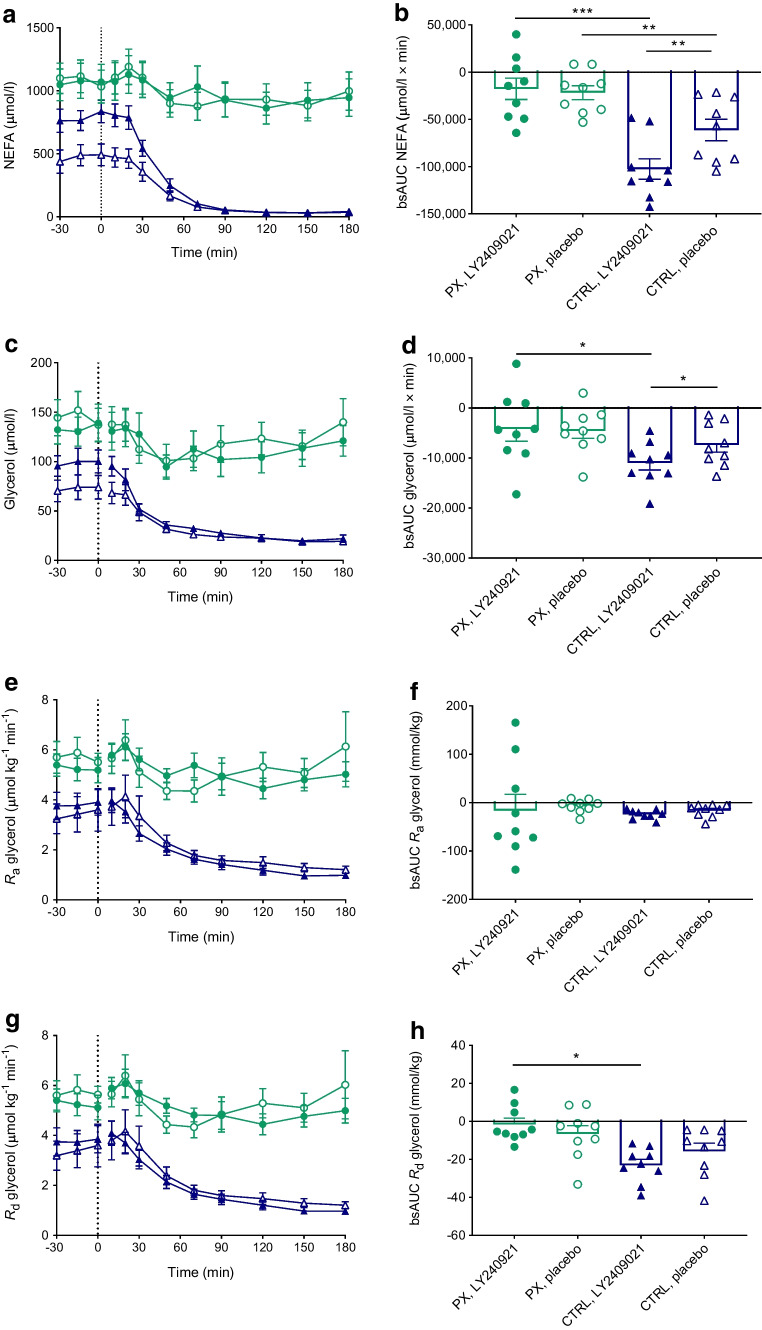
Total plasma NEFA concentration (**a**), total plasma glycerol concentration (**c**), *R*_a_ of glycerol (**e**) and *R*_d_ of glycerol (**g**) during a 75 g OGTT (initiated at time 0 min) in nine totally pancreatectomised participants (PX) (green circles) and nine control participants (CTRL) (blue triangles) with the GRA LY2409021 (filled symbols) or placebo (open symbols). bsAUCs (**b**, **d**, **f, h**) are also shown. Data are shown as means ± SEM. *p≤0.05, **p<0.01, ***p<0.001

On the placebo day, fasting NEFA concentrations were higher in the pancreatectomy group than in the control group (1082 [118] vs 482 [85] pmol/l, *p*<0.001) (Fig. 4a). There was no difference in NEFA concentrations between study days in the pancreatectomy group in the fasting state and OGTT caused a slight suppression of NEFA concentrations after 30 min on both study days. In the control group, significantly higher fasting concentrations of NEFA were observed with LY2409021 compared with placebo (787 [83] vs 482 [85] pmol/l, *p*=0.004). OGTT caused a rapid suppression of NEFA concentrations to similarly low levels on the two study days. There was no difference between study days in fasting concentrations of triglycerides or total, HDL-, LDL- or VLDL-cholesterol in any of the groups and the OGTT did not affect the levels of these circulating lipids (ESM Fig. 2).

### Glycerol kinetics

Fasting concentrations of glycerol on the placebo days were higher in the pancreatectomy group compared with the control group (144 [14] vs 72.8 [10] µmol/l, *p*=0.003) (Fig. 4c). There was neither a significant difference in fasting concentrations of glycerol with LY2409021 compared with placebo in the pancreatectomy group (134 [14] vs 144 [14] µmol/l, *p*=0.461) nor in the control group (98.1 [9.4] vs 72.8 [9.9] µmol/l, *p*=0.053). There was no difference in glycerol kinetics between study days in either of the two groups (Fig. 4e–h).

### Amino acids

**Fig. 5:**
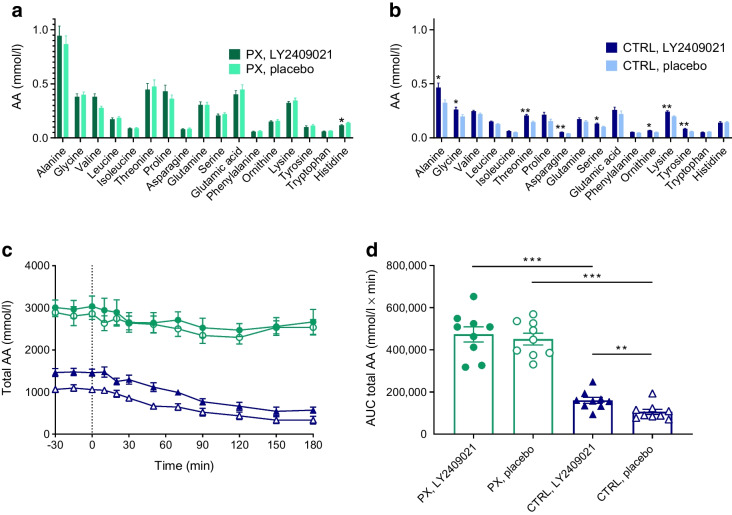
(**a**, **b**) Fasting plasma concentrations of amino acids (AA) in the pancreatectomy group (**a**) and the control group (**b**). (**c**, **d**) Time course of total AA concentrations (**c**) during a 75 g OGTT (initiated at time 0 min) in nine totally pancreatectomised participants (PX) (green bars and circles) and nine control participants (CTRL) (blue bars and triangles) with the GRA LY2409021 (filled symbols) or placebo (open symbols), with the AUC for total AA (**d**) also shown. Data are shown as means SEM. *p≤0.05, **p<0.01, ***p<0.001

Fasting concentrations of individual amino acids and total amino acids during the OGTT were generally higher in the pancreatectomy group than in the control group (Fig. 5a, b). In the pancreatectomy group, there was no difference in fasting concentrations of individual amino acids between study days. Nor was there any difference in total amino acids in the fasting state or during the OGTT. In the control group, fasting levels of individual as well as total amino acids were higher on the day with LY2409021, as were the total amino acids during the OGTT (Fig. 5c, d). The largest increase was found among the amino acids threonine, asparagine, tyrosine and also lysine, with an elevation of 1.4 to 1.5 times basal concentrations. During the OGTT, the concentration of total amino acids (assessed by AUC) was higher with LY2409021 than with placebo in the control group (160,254 [14,304] vs 105,672 [12,462] mmol/l × min, *p*=0.007) (Fig. 5c, d).

### Incretin hormones

**Fig. 6:**
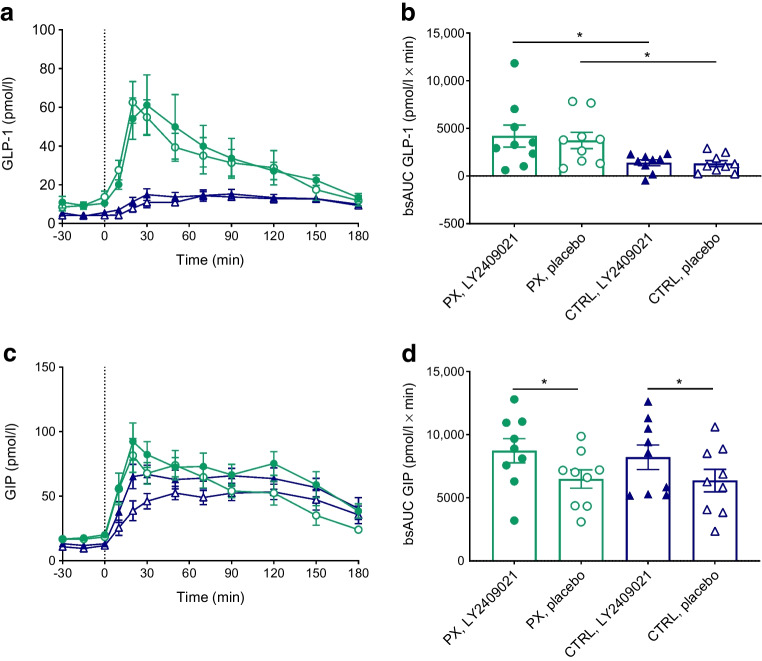
Plasma concentrations of the incretin hormones GLP-1 (**a**) and GIP (**c**) during a 75 g OGTT (initiated at time 0 min) in nine totally pancreatectomised participants (PX) (green circles) and nine control participants (CTRL) (blue triangles) with the GRA LY2409021 (filled symbols) or placebo (open symbols). bsAUCs (**b**, **d**) are also shown. Data are shown as means ± SEM. *p≤0.05

Compared with the control group, the pancreatectomy group exhibited higher fasting plasma GLP-1 (10.4 [1.9] vs 4.2 [0.9] pmol/l, *p*=0.007) and GIP concentrations (17.2 [1.5] vs 10.6 [1.4] pmol/l, *p*=0.006) on the placebo day (Table 3 and Fig. 6a, c). In the pancreatectomy group, the OGTT caused an almost sixfold increase in GLP-1 concentrations, with no difference between study days (*p*=0.823); GIP concentrations showed a fourfold increase and bsAUC was higher with LY2409021 than with placebo (8718 [956] vs 6485 [729] pmol/l × min, *p*=0.024). LY2409021 did not affect fasting or post-OGTT GLP-1 concentrations in the control group whereas the GIP response to OGTT was greater with LY2409021 than with placebo (8209 [973] vs 6361 [893] pmol/l × min, *p*=0.016).

### BP, appetite sensations, food intake, gastric emptying, diuresis and REE

There were no differences in the secondary endpoints BP, appetite sensations, food intake, diuresis and REE after intake of LY2409021 vs placebo in any of the groups. For details, see ESM Results, ESM Table 2, ESM Figs 3 and 4.

## Discussion

In this randomised, double-blind, placebo-controlled, crossover study, we evaluated the metabolic effects of the GRA LY2409021 during OGTT in totally pancreatectomised individuals and matched healthy control individuals aiming to disclose physiological effects of any extrapancreatic glucagon in totally pancreatectomised individuals. We found that LY2409021 did not influence fasting or postabsorptive concentrations of glucose in totally pancreatectomised individuals. Nor did glycerol kinetics or the higher concentrations of NEFA and amino acids observed in the pancreatectomy group change with LY2409021.

### Glucose metabolism

We and others have previously shown that totally pancreatectomised individuals have lower fasting plasma glucagon concentrations but show increments in circulating glucagon concentrations in response to mixed meal test and oral glucose stimulation, respectively [24, 25]. In contrast, i.v. glucose administration suppressed the already low fasting plasma glucagon levels in these individuals [20]. In the present study, we confirmed the low fasting concentration of extrapancreatic glucagon. However, unlike our previous findings, we did not observe any increases in glucagon concentrations during OGTT in the totally pancreatectomised individuals. The reason for this discrepancy is uncertain. A recent study demonstrated that sandwich ELISAs can exhibit cross-reactions with other proglucagon-derived peptides [26], leading to the development of a new assay protocol for the sandwich ELISA with additional washing steps. This new protocol, used in the present study but not in our previous studies, could explain the discrepancy [20, 25].

In contrast to the small but significant reduction in fasting plasma glucose in the control participants, LY2409021 had no impact on plasma glucose concentrations during fasting in the totally pancreatectomised individuals. Neither did it change OGTT-induced glucose excursions or EGP in these individuals, indicating that any extrapancreatic glucagon is not linked to EGP. Paradoxically, and unexpectedly, we observed higher glucose concentrations during the OGTT with LY2409021 in the control participants. Importantly, recent in vitro studies (performed after the execution of the present study) have shown that LY2409021 not only inhibits the glucagon receptor (IC_50_ 28 nmol/l) but also antagonises the GLP-1 receptor and the GIP receptor, with an IC_50_ of 3.4 µmol/l and 2.0 µmol/l, respectively [26]. Based on the dose of 300 mg LY2409021, we expect a plasma concentration of LY2409021 above the IC_50_ concentrations for the GLP-1 and GIP receptors and, consequently, about 80–85% of the activity of the incretin receptors would be blocked [17, 26, 27]. As the incretin hormones GIP and GLP-1 primarily reduce glucose concentrations in the postprandial state, this cross-reactivity may explain why LY2409021 fails to trigger an expected reduction in the postprandial glucose concentrations and instead results in greater OGTT-induced glucose excursions in healthy control individuals. This was not observed in totally pancreatectomised individuals in whom LY2409021 elicited reduced (insignificantly) post-OGTT glucose excursions. Due to the abovementioned cross-reactivity of LY2409021 with the GIP and the GLP-1 receptor (both heavily expressed in pancreatic beta cells), the effect of LY2409021 on post-OGTT glucose excursions in totally pancreatectomised individuals (with no pancreatic GIP and GLP-1 receptors) may better reflect an effect of any circulating glucagon. Thus, based on the present data, we can neither confirm nor exclude that any extrapancreatic glucagon may affect plasma glucose concentrations during OGTT in totally pancreatectomised individuals. The cross-reactivity of LY2409021 may also explain why the greater post-OGTT GIP response with LY2409021 did not affect insulin secretion (assessed by C-peptide) in the healthy control participants. Taken together, the cross-reactivity of LY2409021 clearly prompts reconsideration of the use of this GRA as a tool to investigate the physiological and pathophysiological implications of glucagon. Another possible explanation of the paradoxical increase in post-OGTT glucose excursions in our healthy control participants during glucagon receptor antagonism is the significantly increased rate of gastric emptying observed in the initial phase of the OGTT on the day with LY2409021 in this group. This corresponded to twice as much glucose being emptied from the stomach into the small intestine after 20 min as compared with the placebo day. Exogenous glucagon has previously been shown to reduce gastric emptying [28]. Acceleration of gastric emptying was, nevertheless, not seen in recent studies with LY2409021 in healthy individuals and individuals with type 2 diabetes [27]. No effect of LY2409021 on gastric emptying in totally pancreatectomised individuals was observed.

### Lipid metabolism

Pancreatectomy is associated with accumulation of fat in the liver [29–32], which has been proposed to occur as a consequence of pancreatic glucagon deficiency and ensuing lack of glucagon-induced lipolysis, fatty acid oxidation and inhibition of lipogenesis [8]. In the present study, we observed a twofold higher concentration of NEFA during fasting in the totally pancreatectomised individuals compared with healthy control individuals (Fig. 4a), likely reflecting the insulin-deficient state of these individuals and ensuing lack of insulin-induced inhibition of peripheral lipolysis [33]. Glucagon is known to stimulate oxidation of fatty acids in the liver [33] and we could not detect any effect of LY2409021 on glycerol tracer or NEFA concentrations in the totally pancreatectomised individuals during OGTT (Figs 3a, 4a, c, e, g). In the control participants, concentrations of NEFA were higher after intake of LY2409021 (Fig. 4a). This may relate to the GRA-induced processes causing accumulation of fat in the liver as previously observed with long-term LY2409021 treatment [34] but, perhaps more likely, it may reflect the lower fasting plasma glucose concentrations and the ensuing diminished insulin secretion (as assessed by C-peptide concentrations) and lesser insulin-mediated inhibition of lipolysis on the day of GRA treatment.

### Amino acid metabolism

Glucagon is the main hormone reducing levels of circulating amino acids via stimulation of gluconeogenesis and ureagenesis [35] and several amino acids are strong stimulators of pancreatic glucagon secretion completing the so-called liver–alpha cell axis [8]. In line with these feedback mechanisms controlling the liver–alpha cell axis, individuals who have undergone total pancreatectomy and are depleted of pancreatic glucagon have elevated concentrations of plasma amino acids [8, 36]. Pancreatectomy-associated hyperaminoacidaemia was confirmed in the present study comparing pancreatectomised individuals with control individuals (Fig. 5a, b) and comprised also the glucagonotropic amino acids, threonine, proline, alanine and tyrosine. This supports the notion that removal of pancreatic glucagon decreases hepatic turnover of amino acids, which in turn causes elevation of plasma amino acids, providing a compensatory glucagonotropic signal [8]. Our results in the control participants nicely demonstrate that disruption of the liver–alpha cell axis using the GRA LY2409021 increases circulating concentrations of amino acids, resulting in glucagon secretion and hyperglucagonaemia (Figs 3b, 5b). However, we found no impact of LY2409021 on fasting amino acid concentrations nor on the concentration of total amino acids during the OGTT in the totally pancreatectomised individuals. These findings do not support a role for extrapancreatic glucagon in amino acid metabolism.

### Strengths and limitations

One clear strength of the present study is the well-characterised totally pancreatectomised individuals specifically recruited and volunteering for the experimental procedures. Another strength is the reduced influence of inter-individual confounding factors due to the double-blind, placebo-controlled, crossover design. Furthermore, we reduced carry-over effects of LY2409021 by randomising the order of interventions with an in-between washout period of 14 days. Unfortunately, we did not achieve complete steady-state according to our tracer-to-tracee ratio at baseline (ESM Fig. 5), which may contribute to the decrease in *R*_a_ of endogenous glucose in the fasting state observed in both the pancreatectomy group and the control group. This did not differ between study days in either group. Another limitation was the unexpected findings of cross-reactivity of the presumed specific GRA LY2409021 with the GIP receptor and the GLP-1 receptor from Hædersdal et al [26], rendering us unable to assess the specific effects of glucagon receptor antagonism, especially during OGTT eliciting robust GIP and GLP-1 responses known to influence pancreatic insulin and glucagon secretion. Additionally, recent studies suggest that when applying antibody-based methods to measure glucagon, including sandwich ELISAs considered to be highly specific, cross-reactivity can occur with other proglucagon-derived peptides containing the glucagon sequence, particularly glicentin and oxyntomodulin [24, 37]. For example, in a small study of four totally pancreatectomised individuals, glucagon concentrations were reported to be below the detection limit when applying LC–high-resolution MS compared with the higher concentrations found with the sandwich ELISA [37]. Whether this relates to insufficient sensitivity of LC-MS or specificity problems with the applied glucagon ELISA is unsettled. However, cross-reactivity appears to pose challenges, especially when evaluating plasma samples from individuals with enhanced secretion of proglucagon-derived peptides from the gut, as is the case for totally pancreatectomised individuals [20]. To mitigate cross-reactivity issues, a new protocol incorporating additional washing steps for the sandwich ELISA has been developed and was implemented in the present study. Whether this new protocol increases specificity at the potential cost of reduced sensitivity is not yet determined. We nevertheless acknowledge that specificity issues with previously employed glucagon ELISAs may have overestimated the levels of postabsorptive glucagon concentrations in totally pancreatectomised individuals previously reported [13, 14].

Owing to the restricted inclusion of pancreatectomised participants, sex distribution has not been integrated into the study design, thus, the distribution of men and women is not equal.

### Conclusion

We could not establish any effect of the GRA LY2409021 in totally pancreatectomised individuals. In the control participants, LY2409021 treatment resulted in higher fasting concentration of glucagon, amino acids and NEFA and provide novel insights into which amino acids are involved in the liver–alpha cell axis. Nevertheless, the recently observed antagonising effect of LY2409021 on GLP-1 and GIP receptors, coupled with uncertainties in glucagon measurements, complicate the interpretation of the present findings.

## Supplementary Information

Below is the link to the electronic supplementary material.

ESM (PDF 1.09 MB)

## Data Availability

The datasets generated during and/or analysed during the current study are available from the corresponding author on request.

## Abbreviations

bsAUC: Baseline-subtracted AUC
EGP: Endogenous glucose production
GIP: Glucose-dependent insulinotropic polypeptide
GLP-1: Glucagon-like peptide 1
GRA: Glucagon receptor antagonist
*R*_a_: Rate of appearance
*R*_d_: Rate of disappearance
REE: Resting energy expenditure

## Authors’ relationships and activities

ABL has served on scientific advisory panels and/or received honoraria or research support from Boehringer Ingelheim, Eli Lilly, Novo Nordisk and Zealand Pharma. JJH has served on advisory boards for Novo Nordisk. JJH and MMR are minority shareholders and cofounders of Antag Therapeutics. NJWA has received research support, personal fees and speaker fees from EvoSep, Mercodia, Novo Nordisk, Merck/MSD, Roche and Boehringer Ingelheim. TV has served on scientific advisory panels, been part of speaker’s bureaus, served as a consultant to and/or received research support from Amgen, AstraZeneca, BMS, Boehringer Ingelheim, Eli Lilly, Gilead, GSK, Novo Nordisk, Carmot/Roche, Regor, Sanofi, Sun Pharmaceuticals and Zealand Pharma. FKK is currently employed by Novo Nordisk, the present work was done independently of Novo Nordisk. The authors declare that there are no other relationships or activities that might bias, or be perceived to bias, their work.
