# Supplementary material for: Using glucagon receptor antagonism to evaluate the physiological effects of extrapancreatic glucagon in totally pancreatectomised individuals: a randomised controlled trial
Source: Diabetologia. 2025 Sep 18;68(12):2807–22. doi: 10.1007/s00125-025-06534-z (PMC12594659; doi:10.1007/s00125-025-06534-z)
Supplement: Supplementary file 1 — ESM (PDF 1.09 MB) [file 125_2025_6534_MOESM1_ESM.pdf]

Electronic supplementary material (ESM)

ESM Methods

Conduct, registration and approvals The study was conducted at the Center for Clinical Metabolic Research, Gentofte Hospital, University of Copenhagen, Hellerup, Denmark, in accordance with the Helsinki Declaration, registered at ClinicalTrials.gov (NCT02944110) and approved by the Ethics Committee of the Capital Region of Denmark (H-15009763).

Study participants We included nine totally pancreatectomised participants (seven men and two women) and nine matched healthy control participants. Due to limited opportunities for inclusion by the small number of available pancreatectomised individuals, sex distribution is not integrated in the study design. The key inclusion criteria for the pancreatectomised individuals were age >18 years, normal fasting plasma glucose and HbA_{1c} between 31-44 mmol/mol (5.0-6.2%), haemoglobin >7.0 mmol/l (men) / >6.5 mmol/l (women) and informed consent. Sex was self-reported by participants, options were woman or man. Key inclusion criteria for the healthy control participants were age >18 years, haemoglobin in the normal range and informed consent. Key exclusion criteria for both groups were age >80 years, inflammatory bowel disease and severe liver and/or kidney disease (ESM Fig. 1). For the total pancreatectomy group, additional exclusion criteria were pancreatectomy within the last 3 months and ongoing chemotherapy or chemotherapy within the last 3 months.

Experimental procedures For bedside measurement of plasma glucose, blood was collected into NaF-coated tubes and centrifuged immediately (7,400 *g*, 2 min, room temperature). Blood for analysis of GIP, GLP-1, non-esterified fatty acids and glucagon was collected in chilled tubes (on ice)

containing EDTA and a specific dipeptidyl peptidase 4 (DPP-4) inhibitor, valine pyrrolidide (0.01 ml 1 mmol/l DPP-4 inhibitor solution (i.e. 4.3 mg valine pyrrolidide in 20 ml sterile water) per ml blood). For analysis of insulin, C-peptide, triglycerides, cholesterols and amino acids, blood was sampled in plain tubes for coagulation (20 min, room temperature). Blood for analysis of paracetamol was collected in chilled Li-heparin tubes. EDTA, plain and Li-heparin tubes were centrifuged for 20 minutes at 1,200 *g* and 4°C. Plasma samples for GIP, GLP-1, amino acids, glucagon and tracer analyses were stored at -20°C, and serum samples for insulin, C-peptide, triglycerides, cholesterols and non-esterified fatty acids analyses at -80°C until batch analysis. Heart rate and blood pressure were measured at time -120 min and every 30 minute until time 150 min. Measurement of REE was performed over 15 minutes at time -90, 30 and 150 min. REE was measured by indirect calorimetry using a tight facemask connected to a calorimeter, measuring the gas exchange breath-by-breath via an O_2 alkali cell and an infrared CO_2 sensor (CCMexpress, Medgraphics, Medical Graphics Corporation, St. Paul, Minnesota, USA) (1–3). At time 0, 30, 60, 90, 120, 150 and 180 min, hunger, satiety, fullness and prospective food consumption was assessed by visual analogue scales (VASs). For evaluation of food intake, subjects were served a standardized *ad libitum* meal consisting of minced meat, pasta, corn, carrots, peppers, cream, and salt and pepper (energy (E)% from carbohydrate: 50%; fat: 37%; protein: 13%) at time 180 min and were instructed to eat as much as possible within a maximum of 30 minutes until comfortable satiated. The subjects also evaluated taste, smell, visual appeal, aftertaste and overall palatability of the meal using VASs.

Biochemical analysis C-peptide concentrations were measured with a two-sited sandwich immunoassay using direct chemiluminescent technology (Siemens Healthcare A/S, Ballerup, Denmark) for the ADVIA Centaur XP, intra-assay CV 19%. Plasma concentrations of triglycerides, cholesterols and paracetamol were analyzed by spectrophotometric methods following hydrolysis and oxidation (Siemens Atellica CH 930). Plasma concentrations of non-esterified fatty acids were

measured with a NEFA-HR kit ref: 434-9795/436-91995 from TriChem (Skanderborg, Denmark). Amino acids were analyzed as previously described (4). Total GIP and total GLP-1 were measured with radioimmunoassays as described in (5,6), respectively. Sensitivity for both assays was below 1 pmol/l, and intra assay coefficient of variation below 10%.

ESM results

VAS, food intake, diuresis and energy expenditure When assessed by VAS, there was no difference in sensation of hunger, satiety, prospective food intake, fullness, composite appetite score (CAS), comfort or level of thirst and nausea between study days in any of the two study groups. Results are shown in ESM Table 2 and ESM Fig. 3 as change from baseline over time. Nor was there any difference in amount of food or water ingested between study days. In the pancreatectomy group diuresis was larger on the day with GRA. Baseline values for REE (KJ/24 hours) on the placebo day were higher in the pancreatectomy group compared to the control group (1,765(81) vs 1,370(68) KJ/24 hours, *P*=0.005). No differences in delta values were evident between the two study days in any of the study groups (time -60 min to 30 min: pancreatectomised patients, *P*=0.542; healthy controls, *P*=0.107).

Blood pressure and pulse Both basal (-120 min to time point 0 min) systolic (*P*=0.045) and diastolic blood pressure (*P*=0.022) was higher on the placebo day in the pancreatectomy group (ESM fig. 4). There was no difference in blood pressure (30 to 150 minutes) during OGTT in this group. In the control group both systolic (*P*=0.022) and diastolic (*P*=0.049) blood pressure was higher during OGTT with LY240921, but basal blood pressure was unchanged.

ESM Table 1

	Totally pancreatectomised participants	Healthy control participants	*p value*
**Glucose**			
Mean baseline (mmol/l)			
LY2409021	11.3(0.9)	4.7(0.1)	*<0.001*
Placebo	12.5(0.8)	5.2(0.1)	*<0.001*
P value	*0.266*	*0.001*	
C_{max} (mmol/l)			
LY2409021	25.4(1.0)	12.6(0.7)	*<0.001*
Placebo	26.5(0.9)	10.3(0.5)	*<0.001*
P value	*0.383*	*0.013*	
T_{max} (min)			
LY2409021	138(7.4)	87.8(7.0)	*<0.001*
Placebo	128(8.7)	72.8(10)	*<0.001*
P value	*0.081*	*0.145*	
AUC (mmol/l × min)			
LY2409021	3,708(156)	1,776(97)	*<0.001*
Placebo	3,984(162)	1,405(79)	*<0.001*
P value	*0.179*	*0.008*	
bsAUC (mmol/l × min)			
LY2409021	1,671(96)	926(92)	*<0.001*
Placebo	1,693(152)	467(72)	*<0.001*
P value	*0.810*	*0.002*	
**C-peptide**			
Mean baseline (nmol/l)			
LY2409021	21.7(5.7)	236(25.5)	*<0.001*
Placebo	20.7(3.4)	370(58.2)	*<0.001*
P value	*0.509*	*0.007*	
C_{max} (nmol/l)			
LY2409021	28(10.4)	3,088(300)	*<0.001*
Placebo	30.2(11.7)	2,842(293)	*<0.001*
P value	*0.318*	*0.242*	
T_{max} (min)			
LY2409021	50(26.5)	131(10.8)	*0.013*
Placebo	33.3(21.3)	112(10.5)	*0.006*
P value	*0.444*	*0.070*	
AUC (nmol l^{-1} min^{-1})			

LY2409021	4.8(1.8)	368(42)	*<0.001*
Placebo	4.9(1.9)	340(39.3)	*<0.001*
P value	*0.199*	*0.286*	
bsAUC (nmol/l × min)			
LY2409021	0.8(0.7)	325(39.1)	*<0.001*
Placebo	1.3(1.2)	273(34.5)	*<0.001*
P value	*0.332*	*0.087*	
**Paracetamol**			
C_{max} (mmol/l)			
LY2409021	0.14(0.0)	0.11(0.0)	
Placebo	0.14(0.0)	0.10(0.0)	
P value	*0.437*	*0.739*	
T_{max} (min)			
LY2409021	114.3(16.8)	153.8(11.2)	
Placebo	94.3(16.2)	150(10.7)	
P value	*0.086*	*0.351*	
AUC (mmol/l × min)			
LY2409021	1.7(0.5)	1.1(0.2)	
Placebo	1.9(0.3)	0.7(0.2)	
P value	*0.609*	*0.002*	
bsAUC (mmol/l × min)			
LY2409021	15.8(2.3)	14.1(1.4)	
Placebo	16.9(2.5)	13.1(1.1)	
P value	*0.094*	*0.045*	
**Glucagon**			
Mean baseline (pmol/l)			
LY2409021	0.8(0.0)	7.4(3.1)	*0.059*
Placebo	0.8(0.0)	1.9(0.4)	*0.031*
P value	*0.256*	*0.079*	
C_{max} (pmol/l)			
LY2409021	0.9 (0.1)	10.6 (4.2)	*0.049*
Placebo	1.4 (0.3)	2.7 (0.6)	*0.058*
P value	*0.082)*	*0.072*	
T_{max} (min)			
LY2409021	-6.7 (13)	-10.0 (6.1)	*0.834*
Placebo	14.4 (17)	12.2 (22)	*0.941*
P value	*0.049*	*0.259*	
AUC (pmol/l × min)			
LY2409021	138 (1.7)	366 (84)	*0.025*

Placebo	155 (9.7)	211 (33)	*0.125*
P value	*0.095*	*0.023*	
bsAUC (pmol/l × min)			
LY2409021	-3.3 (3.0)	-988 (482)	*0.074*
Placebo	18.4 (8.9)	-129 (51)	*0.031*
P value	*0.057*	*0.099*	
**GLP-1**			
Mean baseline (pmol/l)			
LY2409021	10.2(2.0)	4.9(0.7)	*0.025*
Placebo	10.4(1.9)	4.2(0.9)	*0.007*
P value	*0.946*	*0.487*	
C_{max} (pmol/l)			
LY2409021	73.1(15)	19.0(2.5)	0.003
Placebo	75.8(10)	18.0(1.9)	*<0.001*
P value	*0.823*	*0.709*	
T_{max} (min)			
LY2409021	42.2(10)	65.6(18)	*0.292*
Placebo	24.4(4)	83.3(15)	*0.002*
P value	*0.180*	*0.520*	
AUC (pmol/l × min)			
LY2409021	6,030(1,115)	2,288(300)	*0.005*
Placebo	5,610(865)	2,053(252)	*0.001*
P value	*0.497*	*0.285*	
bsAUC (pmol/l × min)			
LY2409021	4,191(1,157)	1,395(319)	*0.033*
Placebo	3,730(855)	1,306(316)	*0.017*
P value	*0.646*	*0.810*	
**GIP**			
Mean baseline (pmol/l)			
LY2409021	18.2(1.8)	12.7(1.3)	*0.025*
Placebo	17.2(1.5)	10.6(1.4)	*0.006*
P value	*0.610*	*0.303*	
C_{max} (pmol/l)			
LY2409021	103(10)	78.0(8.4)	*0.086*
Placebo	96.2(11)	60.3(6.1)	*0.016*
P value	*0.415*	*0.006*	
T_{max} (min)			
LY2409021	45.6(11)	84.4(15)	*0.065*
Placebo	51.5(10)	78.9(13)	*0.119*

P value	*0.733*	*0.845*	
AUC (pmol/l × min)			
LY2409021	11,988(1,150)	10,495(1,094)	*0.361*
Placebo	9,586(919)	8,274(921)	*0.329*
P value	*0.025*	*0.005*	
bsAUC (pmol/l × min)			
LY2409021	8,718(956)	8,209(973)	*0.714*
Placebo	6,485(729)	6,361(893)	*0.915*
P value	*0.024*	*0.016*	

Plasma/serum concentrations of glucose, C-peptide, paracetamol , glucagon, glucagon-like peptide 1 (GLP-1) and glucose-dependent insulinotropic polypeptide (GIP) during a 3-hour 75g-oral glucose tolerance test (OGTT) with 1.5 g paracetamol in 9 totally pancreatectomised participants and 9 control participants with LY2409021 or placebo. Data are mean with standard error of the mean in brackets. Statistical analysis was performed by two-sample student's *t* test (two-tailed), paired within groups and unpaired between groups. AUC, area under curve; bsAUC, baseline-subtracted AUC; C_{max}, maximum serum/plasma concentration; T_{max},, median time for maximum drug concentration.

ESM Table 2

	PX participants LY2409021	PX participants placebo	CTRL participants LY2409021	CTRL participants placebo
Hunger (mm × min)	1,100(1,019)	2,063(1,074)	940(909)	-63(666)
Satiety (mm × min)	-750(659)	-1,125(603)	277(952)	778(779)
Fullness (mm × min)	807(836)	298(666)	2,312(836)	2,475(977)
Prospective food intake (mm × min)	232(658)	143(356)	-603(1,009)	-520(716)
Composite appetite score (mm × min)	319(550)	758(487)	-563(701)	-959(524)
Nausea (mm × min)	-910(602)	-845(607)	-472(656)	-302(311)
Thirst (mm × min)	923(1,078)	-302(486)	670(807)	527(741)
Comfort (mm × min)	-125(747)	327(670)	-247(428)	-627(407)
Food intake (g)	435(71)	515(94)	326(66)	413(82)
Food intake (Kcal)				
Water intake (g)	556(73)	545(83)	478(90)	459(101)
Urine (ml)*	343(71)* (*P*=0.015)	486(82)	199(33)	225(45)

Visual analog score (VAS) values are mean baseline-subtracted area under curve (bsAUC ± SEM) (measured in millimeters (mm)) as response over time during a 75g-OGTT with LY2409021 or placebo in totally pancreatectomised participants (PX) (n=9) and healthy control participants (CTRL) (n=9). Food intake (g), energy (kcal), water intake (g) and diuresis (ml) values are mean (SEM). Asterisks (*$P<0.05$) indicate significant difference between experimental days within groups.

ESM Figure 1. Flowchart

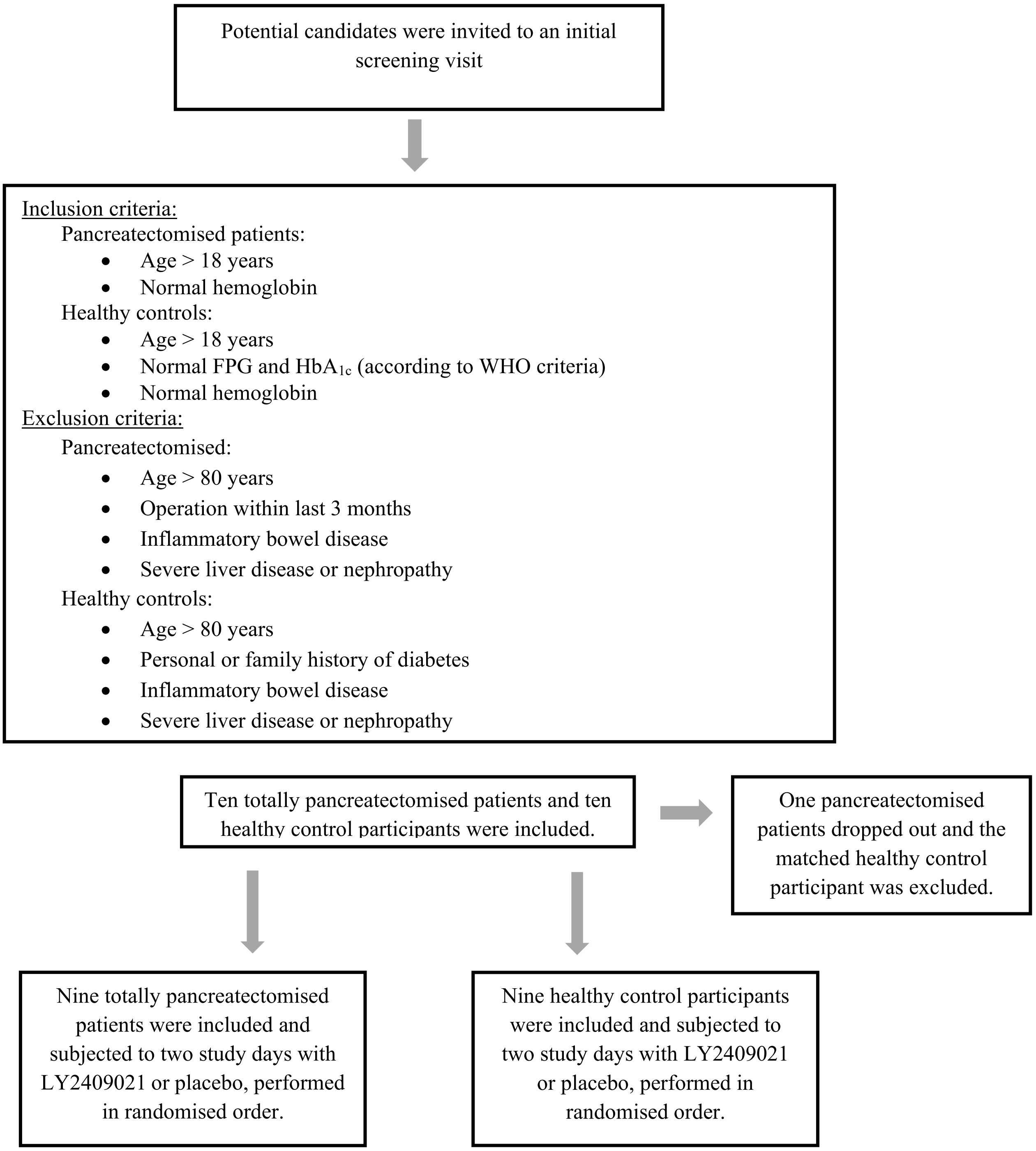

Flowchart depicting the recruitment process including the most relevant inclusion and exclusion criteria. FPG, fasting plasma glucose; WHO, World Health Organization

ESM Figure 2. Lipid profile

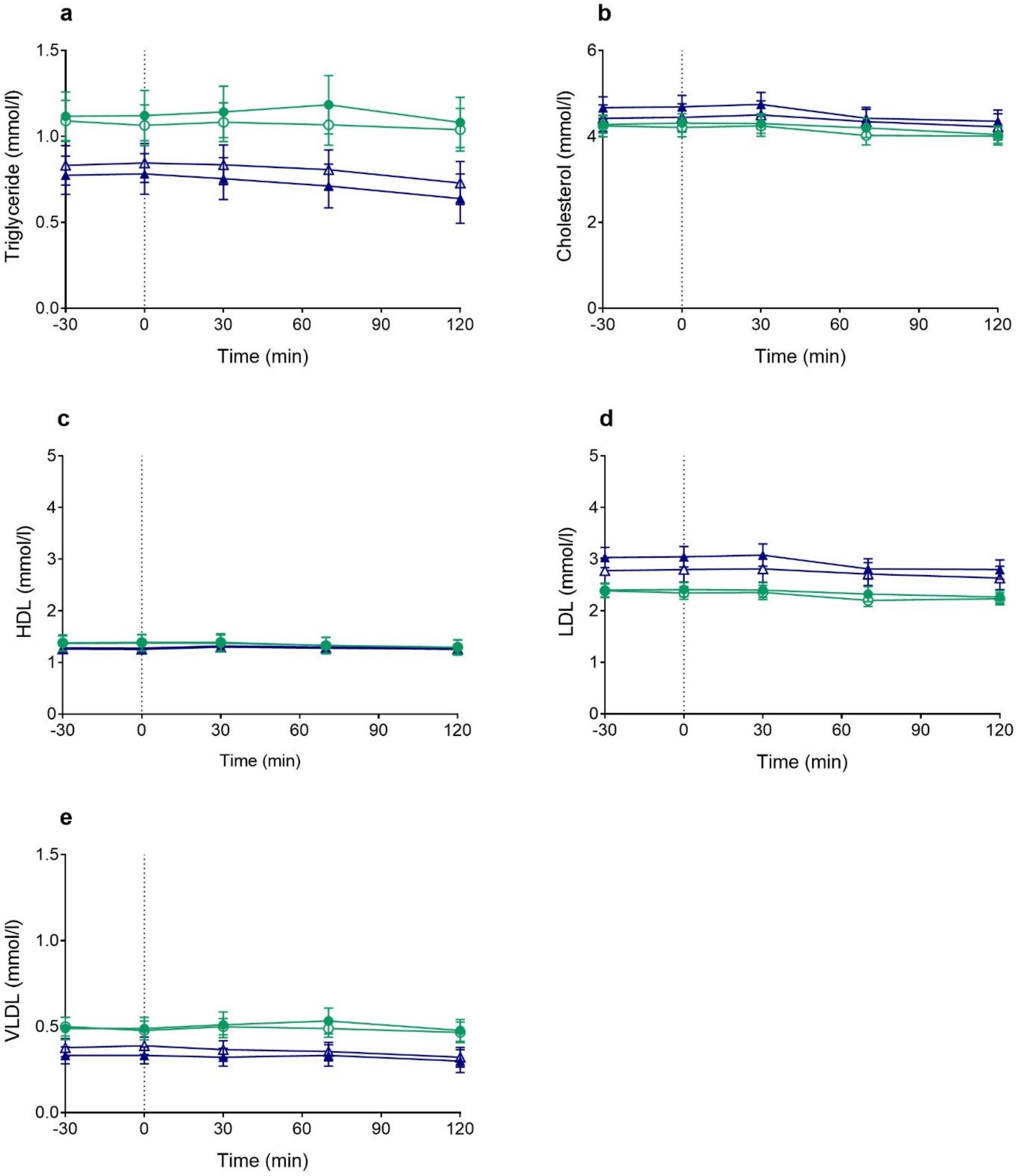

Plasma concentrations of triglyceride (a), cholesterol (b), HDL (c), LDL (d) and VLDL (e) during a 75g-OGTT (ingested at time 0 min) in 9 totally pancreatectomised participants (PX) (green curves/circles) and 9 healthy control participants (CTRL) (blue curves/triangles) with LY2409021(filled symbols) or placebo (open symbols). For statistical differences, please refer to the text. Data are shown as mean ± SEM.

ESM Figure 3. Visual analogue score values.

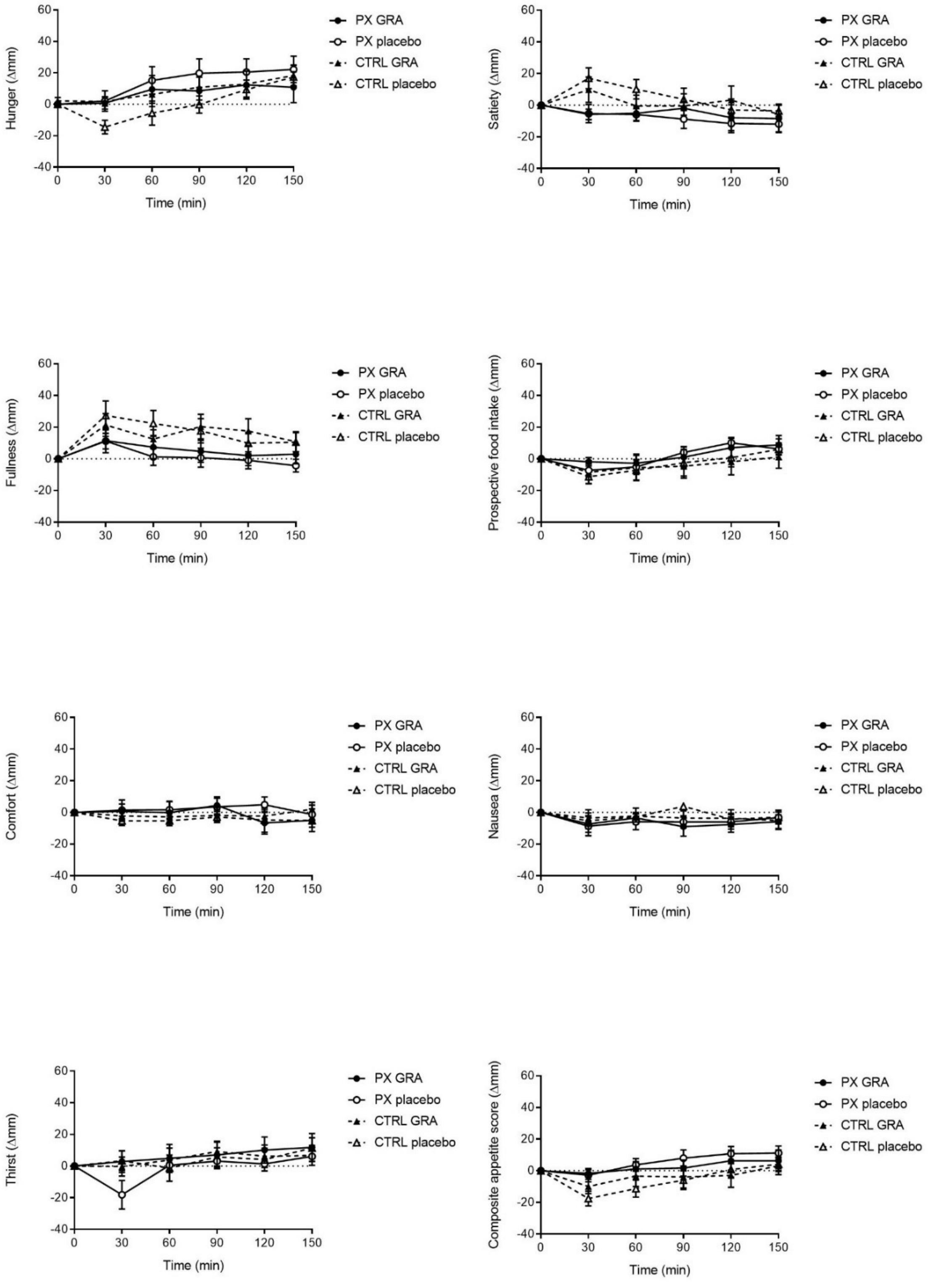

Sensations of appetite, satiety, thirst and well-being. Changes in visual analog score values (VAS) (baseline-subtracted and measured in millimeters (mm)) as response over time during a 75g-OGTT

in totally pancreatectomised participants (PX) (circles, full line) and healthy control participants (CTRL) (triangles, dottet line) with GRA (closed symbols) or placebo (open symbols). Composite appetite score (CAS) was calculated, as [(hunger + prospective food consumption + (100 – satiety) + (100 – fullness)) / 4]. Data are shown as mean ± SEM.

ESM Figure 4. Blood pressure and pulse

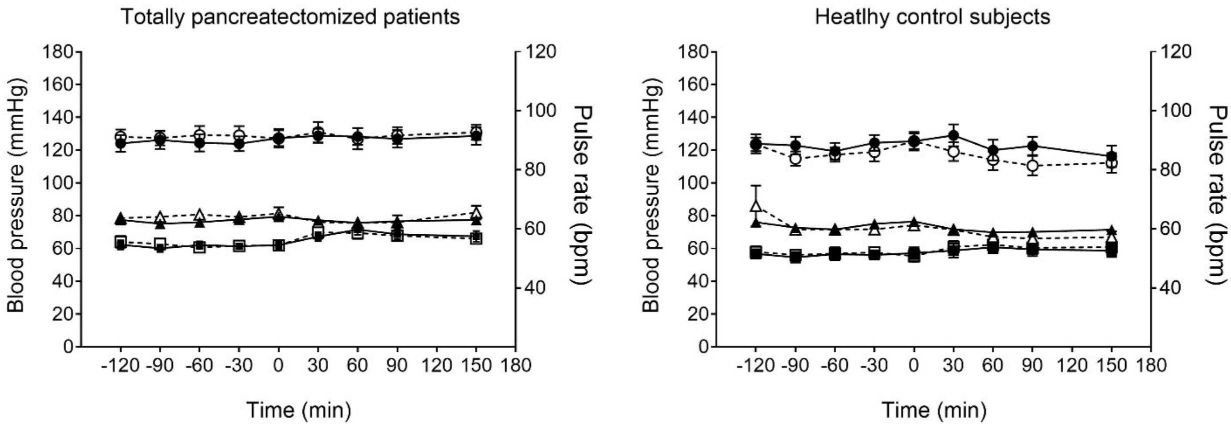

Blood pressure and pulse. Systolic blood pressure (bp) (circles) and diastolic bp (triangles) and pulse rate (squares) during a 75g-OGTT in totally pancreatectomised (PX) (a) and healthy control participants (CTRL) (b) during liquid mixed meal test with LY2409021 (full curves, closed symbols) or placebo (dotted curves, open symbols).

ESM Figure 5. Enrichment of isotope-marked glucose

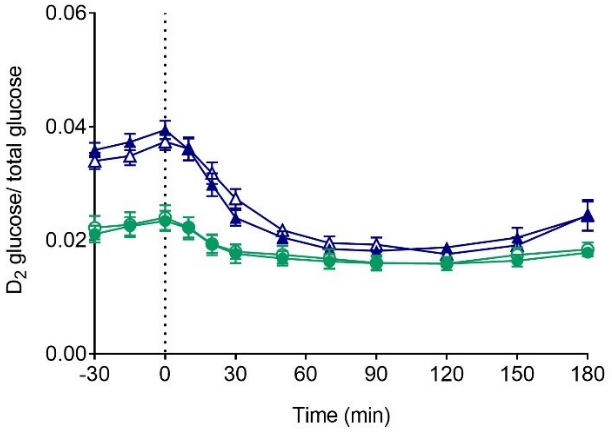

Enrichment of isotope-marked glucose expressed as tracer to tracee ratio during a 75g-OGTT (ingested at time 0 min) in 9 totally pancreatectomised participants (PX) (circles) and 9 healthy control participants (CTRL) (triangles) with LY2409021(filled symbols) or placebo (open symbols). Data are shown as mean ± SEM.

References

1. Ferrannini E. The theoretical bases of indirect calorimetry: A review. Metabolism. 1988;37(3):287–301.

2. Weir J. New methods for calculating metabolic rate with special reference to protein metabolism. J Physiol. 1949 Aug;109(1–2):1–9.

3. Compher C, Frankenfield D, Keim N, Roth-Yousey L, Evidence Analysis Working Group. Best practice methods to apply to measurement of resting metabolic rate in adults: a systematic review. J Am Diet Assoc. 2006 Jun;106(6):881–903.

4. Wewer Albrechtsen NJ, Junker AE, Christensen M, Haedersdal S, Wibrand F, Lund AM, et al. Hyperglucagonemia correlates with plasma levels of non-branched-chain amino acids in patients with liver disease independent of type 2 diabetes. Am J Physiol Gastrointest Liver Physiol. 2018;314(1):G91–6.

5. Ørskov C, Rabenhøj L, Wettergren A, Kofod H, Hoist JJ. Tissue and Plasma Concentrations of Amidated and Glycine-Extended Glucagon-Like Peptide I in Humans. Diabetes. 1994;43:535–74.

6. Lindgren O, Carr RD, Deacon CF, Holst JJ, Pacini G, Mari A, et al. Incretin hormone and insulin responses to oral versus intravenous lipid administration in humans. J Clin Endocrinol Metab. 2011;96(8):2519–24.
